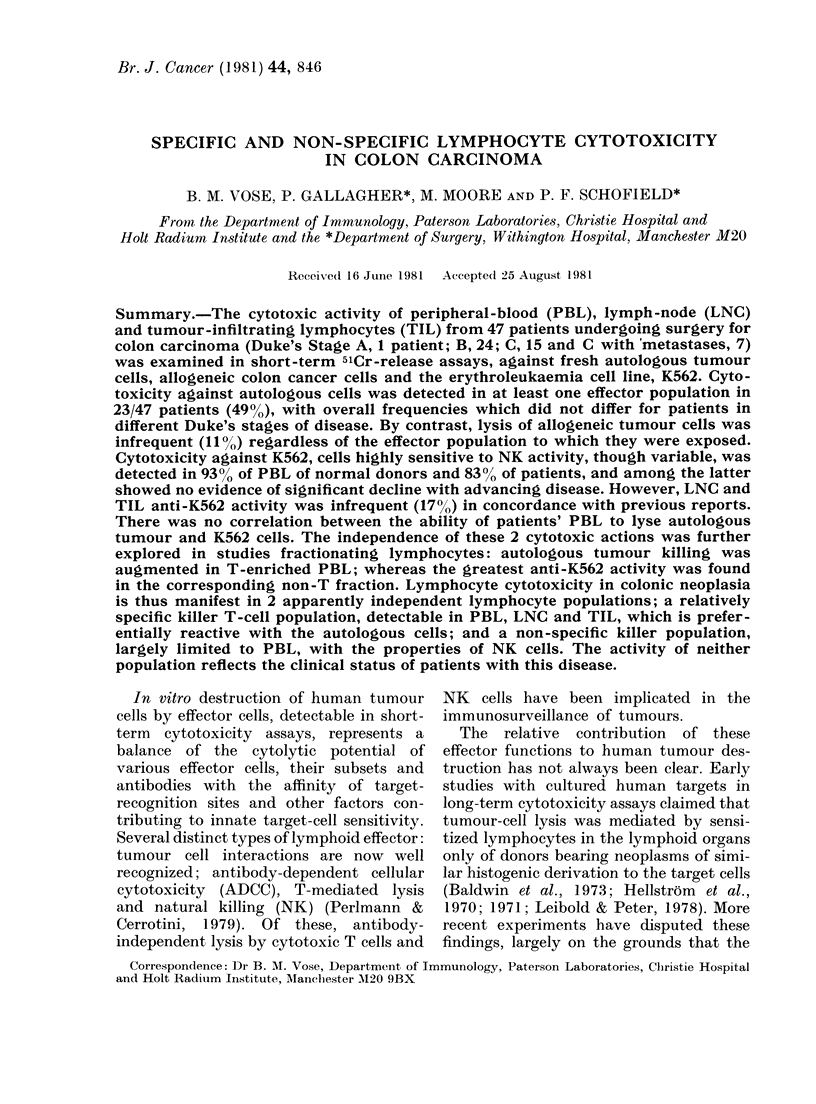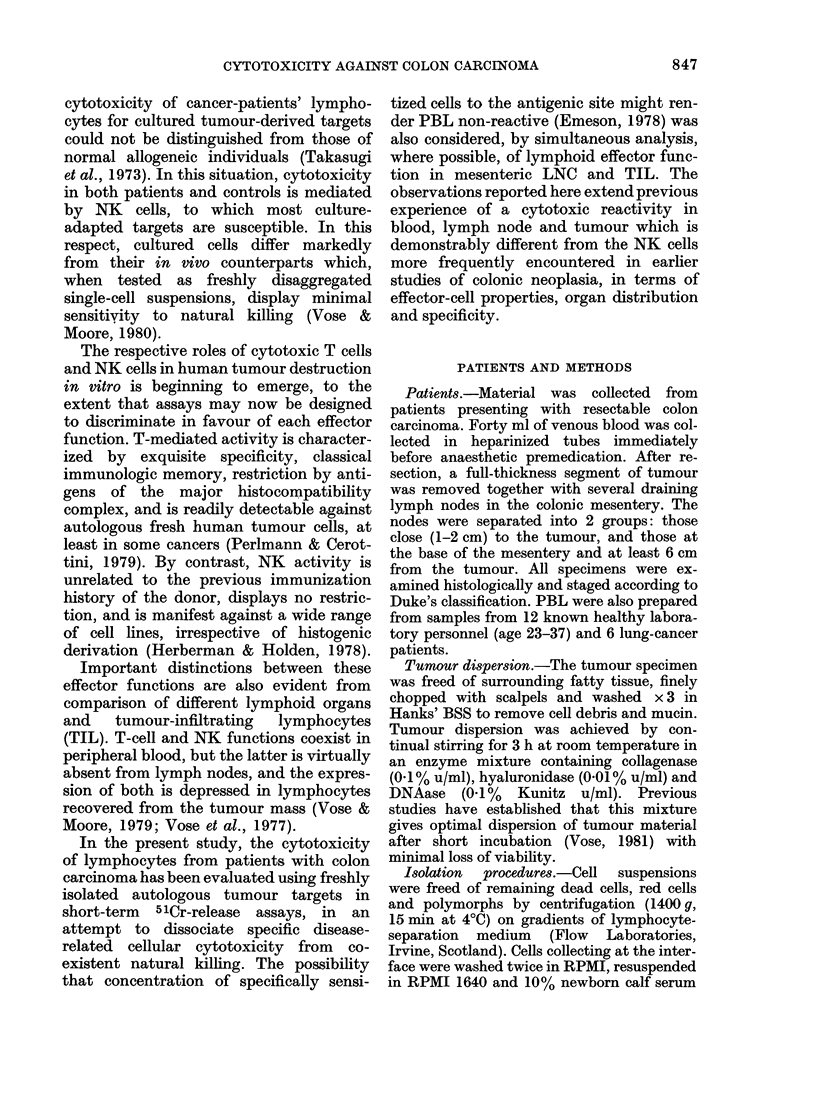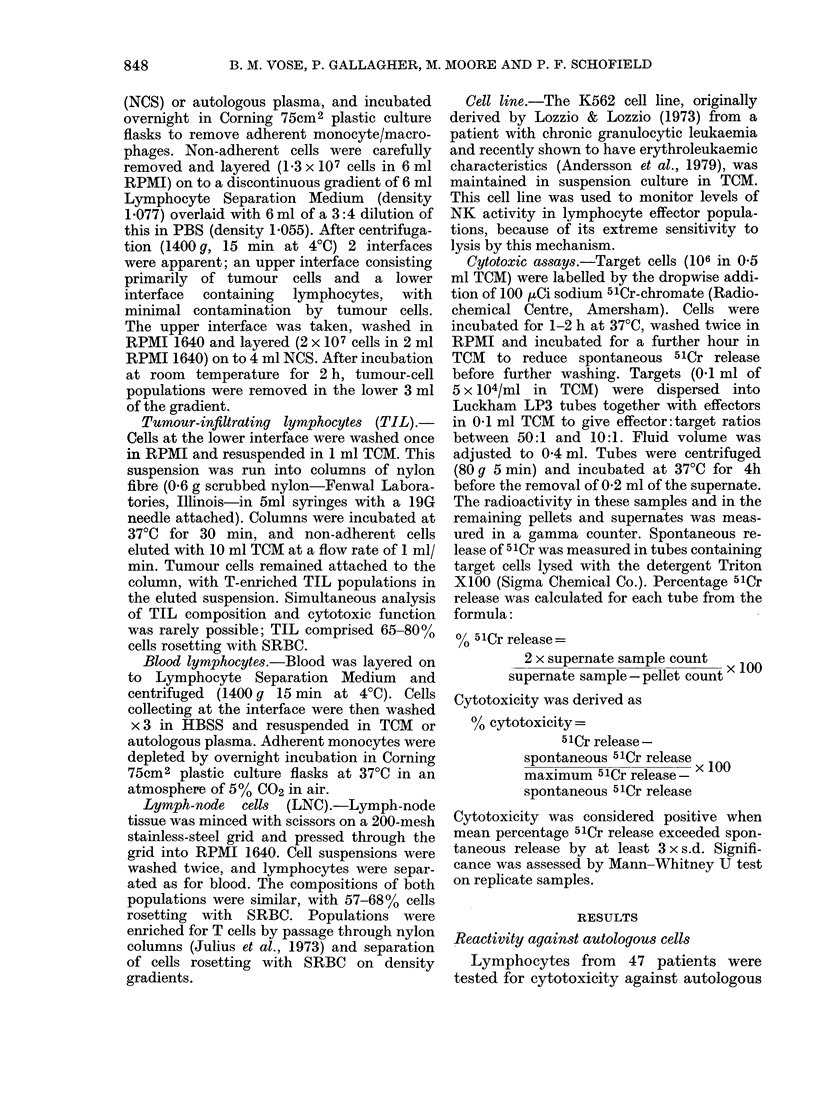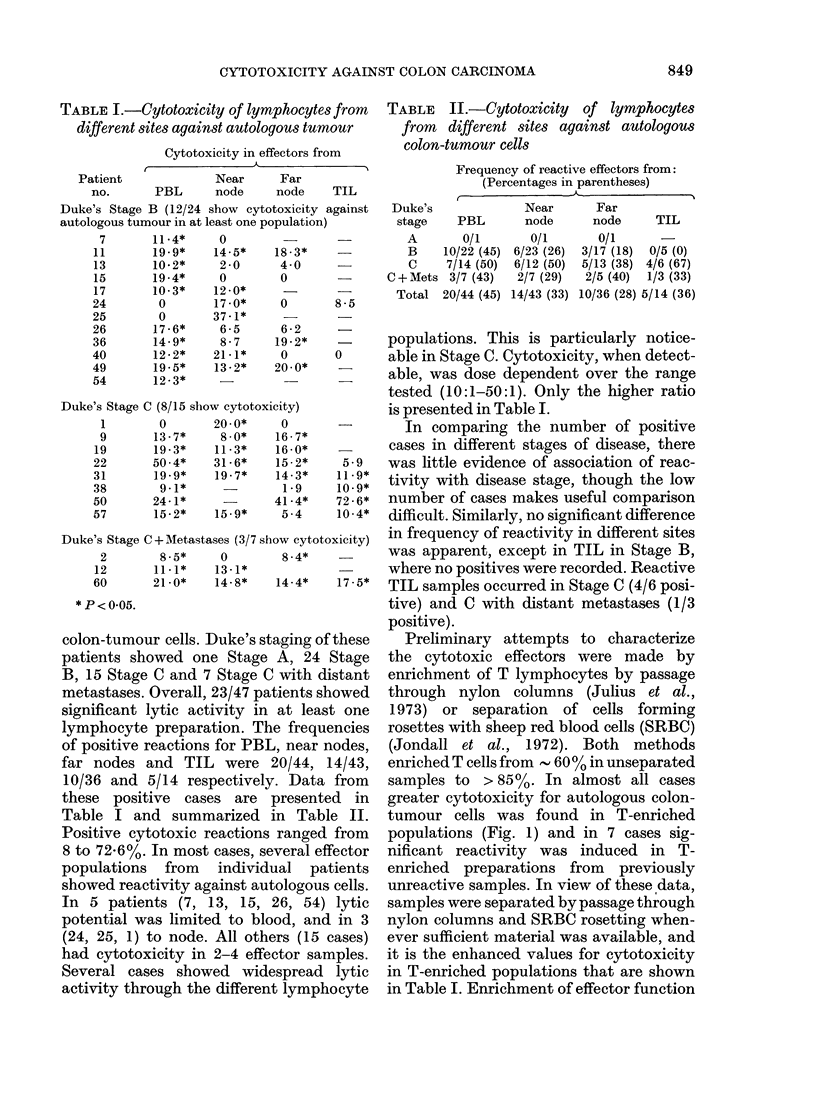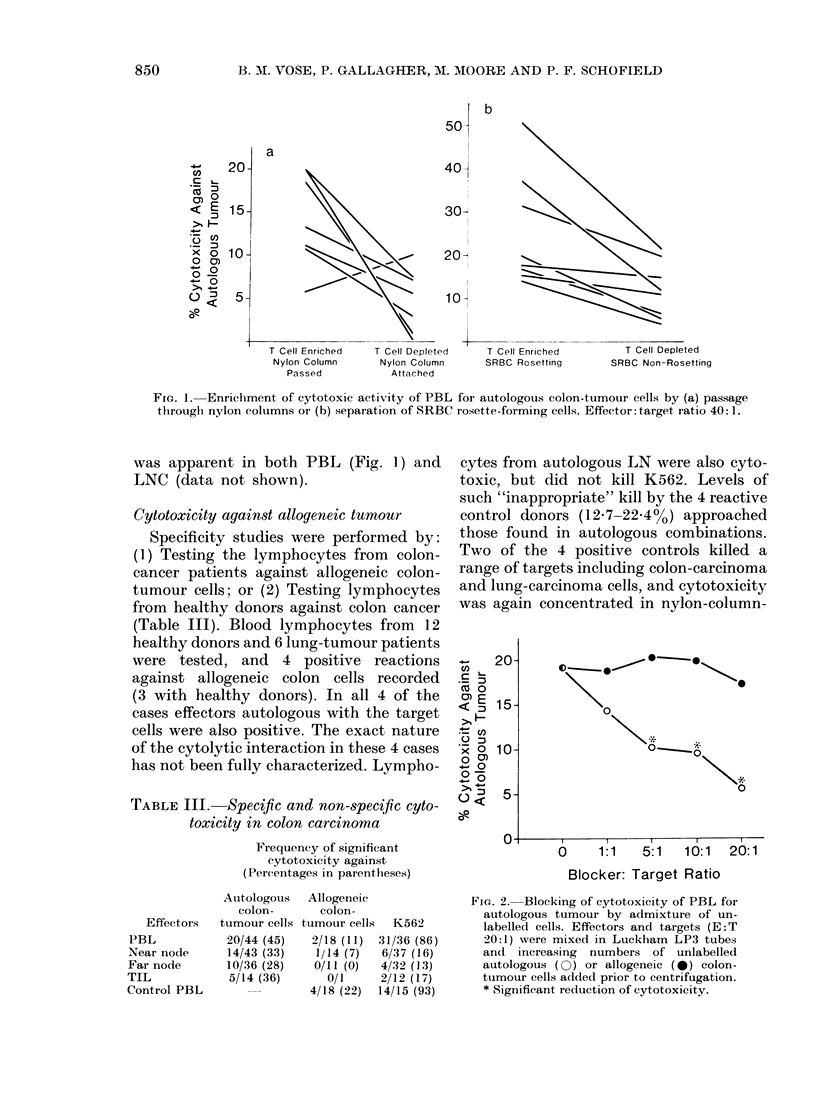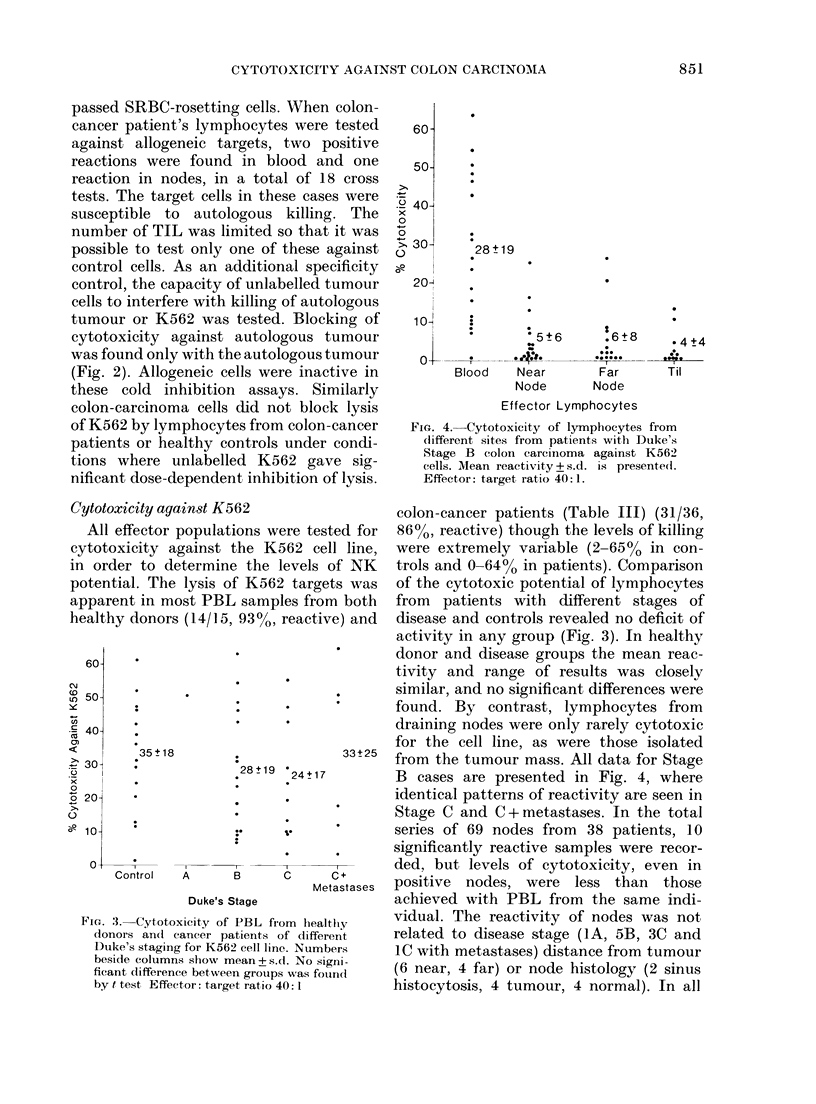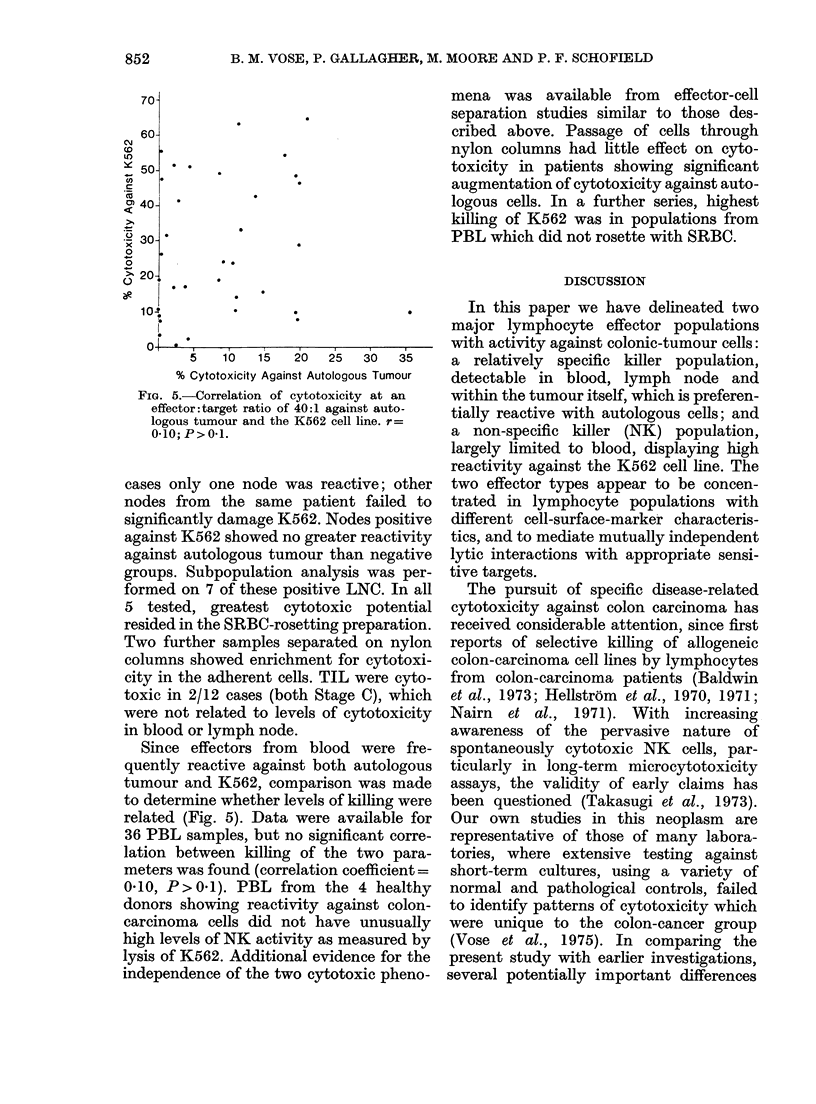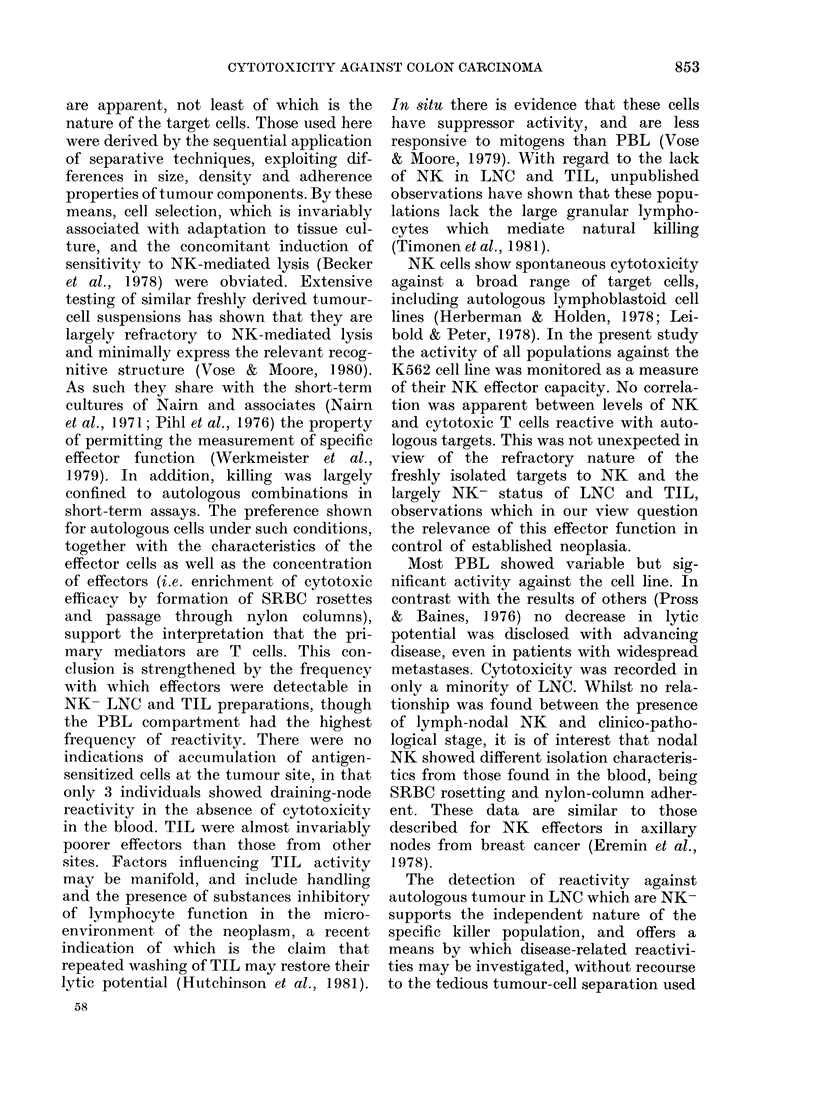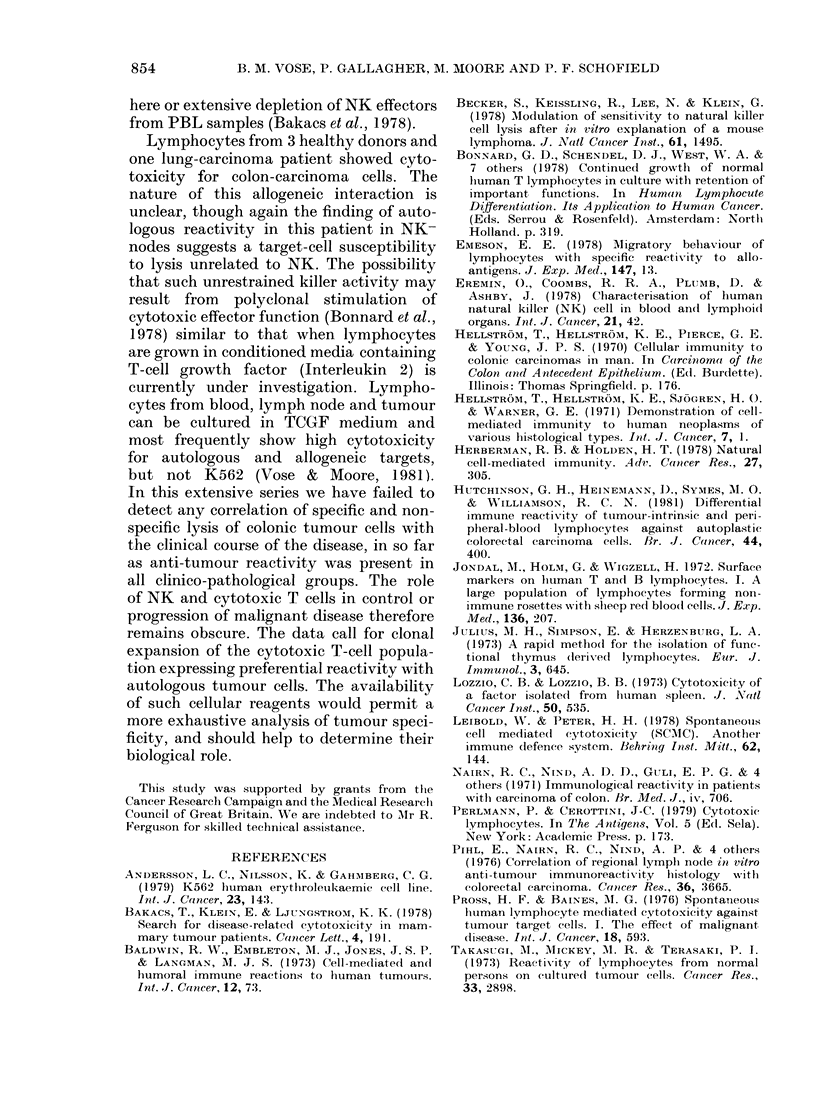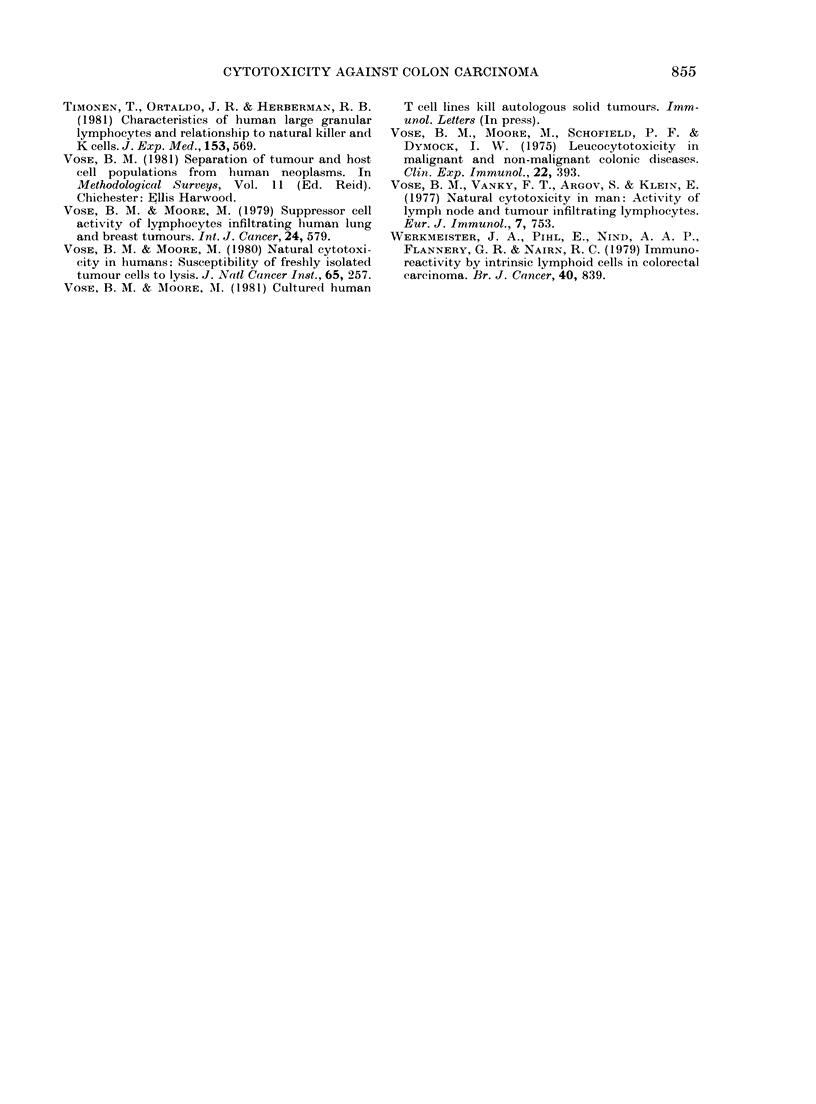# Specific and non-specific lymphocyte cytotoxicity in colon carcinoma.

**DOI:** 10.1038/bjc.1981.283

**Published:** 1981-12

**Authors:** B. M. Vose, P. Gallagher, M. Moore, P. F. Schofield

## Abstract

The cytotoxic activity of peripheral-blood (PBL), lymph-node (LNC) and tumour-infiltrating lymphocytes (TIL) from 47 patients undergoing surgery for colon carcinoma (Duke's Stage A, 1 patient; B, 24; C, 15 and C with metastases, 7) was examined in short-term 51Cr-release assays, against fresh autologous tumour cells, allogeneic colon cancer cells and the erythroleukaemia cell line, K562. Cytotoxicity against autologous cells was detected in at least one effector population in 23/47 patients (49%), with overall frequencies which did not differ for patients in different Duke's stages of disease. By contrast, lysis of allogeneic tumour cells was infrequent (11%) regardless of the effector population to which they were exposed. Cytotoxicity against K562, cells highly sensitive to NK activity, though variable, was detected in 93% of PBL of normal donors and 83% of patients, and among the latter showed no evidence of significant decline with advancing disease. However, LNC and TIL anti-K562 activity was infrequent (17%) in concordance with previous reports. There was no correlation between the ability of patients' PBL to lyse autologous tumour and K562 cells. The independence of these 2 cytotoxic actions was further explored in studies fractionating lymphocytes: autologous tumour killing was augmented in T-enriched PBL; whereas the greatest anti-K562 activity was found in the corresponding non-T fraction. Lymphocyte cytotoxicity in colonic neoplasia is thus manifest in 2 apparently independent lymphocyte populations; a relatively specific killer T-cell population, detectable in PBL, LNC and TIL, which is preferentially reactive with the autologous cells; and a non-specific killer population, largely limited to PBL, with the properties of NK cells. The activity of neither population reflects the clinical status of patients with this disease.


*Br. J. Cancer* (1981) **44**, 846

### SPECIFIC AND NON-SPECIFIC LYMPHOCYTE CYTOTOXICITY IN COLON CARCINOMA

B. M. VOSE, P. GALLAGHER*, M. MOORE AND P. F. SCHOFIELD*

*From the Department of Immunology, Paterson Laboratories, Christie Hospital and Holt Radium Institute and the *Department of Surgery, Withington Hospital, Manchester M20*

Received 16 June 1981 Accepted 25 August 1981

**Summary.—The cytotoxic activity of peripheral-blood (PBL), lymph-node (LNC) and tumour-infiltrating lymphocytes (TIL) from 47 patients undergoing surgery for colon carcinoma (Duke's Stage A, 1 patient; B, 24; C, 15 and C with metastases, 7) was examined in short-term $^{51}$Cr-release assays, against fresh autologous tumour cells, allogeneic colon cancer cells and the erythroleukaemia cell line, K562. Cytotoxicity against autologous cells was detected in at least one effector population in 23/47 patients (49%), with overall frequencies which did not differ for patients in different Duke's stages of disease. By contrast, lysis of allogeneic tumour cells was infrequent (11%) regardless of the effector population to which they were exposed. Cytotoxicity against K562, cells highly sensitive to NK activity, though variable, was detected in 93% of PBL of normal donors and 83% of patients, and among the latter showed no evidence of significant decline with advancing disease. However, LNC and TIL anti-K562 activity was infrequent (17%) in concordance with previous reports. There was no correlation between the ability of patients' PBL to lyse autologous tumour and K562 cells. The independence of these 2 cytotoxic actions was further explored in studies fractionating lymphocytes: autologous tumour killing was augmented in T-enriched PBL; whereas the greatest anti-K562 activity was found in the corresponding non-T fraction. Lymphocyte cytotoxicity in colonic neoplasia is thus manifest in 2 apparently independent lymphocyte populations; a relatively specific killer T-cell population, detectable in PBL, LNC and TIL, which is preferentially reactive with the autologous cells; and a non-specific killer population, largely limited to PBL, with the properties of NK cells. The activity of neither population reflects the clinical status of patients with this disease.**

*In vitro* destruction of human tumour cells by effector cells, detectable in short-term cytotoxicity assays, represents a balance of the cytolytic potential of various effector cells, their subsets and antibodies with the affinity of target-recognition sites and other factors contributing to innate target-cell sensitivity. Several distinct types of lymphoid effector: tumour cell interactions are now well recognized; antibody-dependent cellular cytotoxicity (ADCC), T-mediated lysis and natural killing (NK) (Perlmann & Cerrotini, 1979). Of these, antibody-independent lysis by cytotoxic T cells and NK cells have been implicated in the immunosurveillance of tumours.

The relative contribution of these effector functions to human tumour destruction has not always been clear. Early studies with cultured human targets in long-term cytotoxicity assays claimed that tumour-cell lysis was mediated by sensitized lymphocytes in the lymphoid organs only of donors bearing neoplasms of similar histogenic derivation to the target cells (Baldwin *et al.*, 1973; Hellström *et al.*, 1970; 1971; Leibold & Peter, 1978). More recent experiments have disputed these findings, largely on the grounds that the

Correspondence: Dr B. M. Vose, Department of Immunology, Paterson Laboratories, Christie Hospital and Holt Radium Institute, Manchester M20 9BX

cytotoxicity of cancer-patients' lymphocytes for cultured tumour-derived targets could not be distinguished from those of normal allogeneic individuals (Takasugi *et al.*, 1973). In this situation, cytotoxicity in both patients and controls is mediated by NK cells, to which most culture-adapted targets are susceptible. In this respect, cultured cells differ markedly from their *in vivo* counterparts which, when tested as freshly disaggregated single-cell suspensions, display minimal sensitivity to natural killing (Vose & Moore, 1980).

The respective roles of cytotoxic T cells and NK cells in human tumour destruction *in vitro* is beginning to emerge, to the extent that assays may now be designed to discriminate in favour of each effector function. T-mediated activity is characterized by exquisite specificity, classical immunologic memory, restriction by antigens of the major histocompatibility complex, and is readily detectable against autologous fresh human tumour cells, at least in some cancers (Perlmann & Cerottini, 1979). By contrast, NK activity is unrelated to the previous immunization history of the donor, displays no restriction, and is manifest against a wide range of cell lines, irrespective of histogenic derivation (Herberman & Holden, 1978).

Important distinctions between these effector functions are also evident from comparison of different lymphoid organs and tumour-infiltrating lymphocytes (TIL). T-cell and NK functions coexist in peripheral blood, but the latter is virtually absent from lymph nodes, and the expression of both is depressed in lymphocytes recovered from the tumour mass (Vose & Moore, 1979; Vose *et al.*, 1977).

In the present study, the cytotoxicity of lymphocytes from patients with colon carcinoma has been evaluated using freshly isolated autologous tumour targets in short-term $^{51}Cr$-release assays, in an attempt to dissociate specific disease-related cellular cytotoxicity from co-existent natural killing. The possibility that concentration of specifically sensitized cells to the antigenic site might render PBL non-reactive (Emeson, 1978) was also considered, by simultaneous analysis, where possible, of lymphoid effector function in mesenteric LNC and TIL. The observations reported here extend previous experience of a cytotoxic reactivity in blood, lymph node and tumour which is demonstrably different from the NK cells more frequently encountered in earlier studies of colonic neoplasia, in terms of effector-cell properties, organ distribution and specificity.

### PATIENTS AND METHODS

*Patients.*—Material was collected from patients presenting with resectable colon carcinoma. Forty ml of venous blood was collected in heparinized tubes immediately before anaesthetic premedication. After resection, a full-thickness segment of tumour was removed together with several draining lymph nodes in the colonic mesentery. The nodes were separated into 2 groups: those close (1–2 cm) to the tumour, and those at the base of the mesentery and at least 6 cm from the tumour. All specimens were examined histologically and staged according to Duke's classification. PBL were also prepared from samples from 12 known healthy laboratory personnel (age 23–37) and 6 lung-cancer patients.

*Tumour dispersion.*—The tumour specimen was freed of surrounding fatty tissue, finely chopped with scalpels and washed ×3 in Hanks' BSS to remove cell debris and mucin. Tumour dispersion was achieved by continual stirring for 3 h at room temperature in an enzyme mixture containing collagenase (0·1% u/ml), hyaluronidase (0·01% u/ml) and DNAase (0·1% Kunitz u/ml). Previous studies have established that this mixture gives optimal dispersion of tumour material after short incubation (Vose, 1981) with minimal loss of viability.

*Isolation procedures.*—Cell suspensions were freed of remaining dead cells, red cells and polymorphs by centrifugation (1400 *g*, 15 min at 4°C) on gradients of lymphocyte-separation medium (Flow Laboratories, Irvine, Scotland). Cells collecting at the interface were washed twice in RPMI, resuspended in RPMI 1640 and 10% newborn calf serum

(NCS) or autologous plasma, and incubated overnight in Corning 75cm$^2$ plastic culture flasks to remove adherent monocyte/macrophages. Non-adherent cells were carefully removed and layered ($1{\cdot}3 \times 10^7$ cells in 6 ml RPMI) on to a discontinuous gradient of 6 ml Lymphocyte Separation Medium (density 1·077) overlaid with 6 ml of a 3:4 dilution of this in PBS (density 1·055). After centrifugation (1400 *g*, 15 min at 4°C) 2 interfaces were apparent; an upper interface consisting primarily of tumour cells and a lower interface containing lymphocytes, with minimal contamination by tumour cells. The upper interface was taken, washed in RPMI 1640 and layered ($2 \times 10^7$ cells in 2 ml RPMI 1640) on to 4 ml NCS. After incubation at room temperature for 2 h, tumour-cell populations were removed in the lower 3 ml of the gradient.

*Tumour-infiltrating lymphocytes* (*TIL*).—Cells at the lower interface were washed once in RPMI and resuspended in 1 ml TCM. This suspension was run into columns of nylon fibre (0·6 g scrubbed nylon—Fenwal Laboratories, Illinois—in 5ml syringes with a 19G needle attached). Columns were incubated at 37°C for 30 min, and non-adherent cells eluted with 10 ml TCM at a flow rate of 1 ml/min. Tumour cells remained attached to the column, with T-enriched TIL populations in the eluted suspension. Simultaneous analysis of TIL composition and cytotoxic function was rarely possible; TIL comprised 65–80% cells rosetting with SRBC.

*Blood lymphocytes.*—Blood was layered on to Lymphocyte Separation Medium and centrifuged (1400 *g* 15 min at 4°C). Cells collecting at the interface were then washed ×3 in HBSS and resuspended in TCM or autologous plasma. Adherent monocytes were depleted by overnight incubation in Corning 75cm$^2$ plastic culture flasks at 37°C in an atmosphere of 5% $CO_2$ in air.

*Lymph-node cells* (LNC).—Lymph-node tissue was minced with scissors on a 200-mesh stainless-steel grid and pressed through the grid into RPMI 1640. Cell suspensions were washed twice, and lymphocytes were separated as for blood. The compositions of both populations were similar, with 57–68% cells rosetting with SRBC. Populations were enriched for T cells by passage through nylon columns (Julius *et al.*, 1973) and separation of cells rosetting with SRBC on density gradients.

*Cell line.*—The K562 cell line, originally derived by Lozzio & Lozzio (1973) from a patient with chronic granulocytic leukaemia and recently shown to have erythroleukaemic characteristics (Andersson *et al.*, 1979), was maintained in suspension culture in TCM. This cell line was used to monitor levels of NK activity in lymphocyte effector populations, because of its extreme sensitivity to lysis by this mechanism.

*Cytotoxic assays.*—Target cells ($10^6$ in 0·5 ml TCM) were labelled by the dropwise addition of 100 μCi sodium $^{51}$Cr-chromate (Radiochemical Centre, Amersham). Cells were incubated for 1–2 h at 37°C, washed twice in RPMI and incubated for a further hour in TCM to reduce spontaneous $^{51}$Cr release before further washing. Targets (0·1 ml of $5 \times 10^4$/ml in TCM) were dispersed into Luckham LP3 tubes together with effectors in 0·1 ml TCM to give effector:target ratios between 50:1 and 10:1. Fluid volume was adjusted to 0·4 ml. Tubes were centrifuged (80 *g* 5 min) and incubated at 37°C for 4h before the removal of 0·2 ml of the supernate. The radioactivity in these samples and in the remaining pellets and supernates was measured in a gamma counter. Spontaneous release of $^{51}$Cr was measured in tubes containing target cells lysed with the detergent Triton X100 (Sigma Chemical Co.). Percentage $^{51}$Cr release was calculated for each tube from the formula:

$$\%\ {}^{51}\text{Cr release} = \frac{2 \times \text{supernate sample count}}{\text{supernate sample} - \text{pellet count}} \times 100$$

Cytotoxicity was derived as

$$\%\ \text{cytotoxicity} = \frac{{}^{51}\text{Cr release} - \text{spontaneous } {}^{51}\text{Cr release}}{\text{maximum } {}^{51}\text{Cr release} - \text{spontaneous } {}^{51}\text{Cr release}} \times 100$$

Cytotoxicity was considered positive when mean percentage $^{51}$Cr release exceeded spontaneous release by at least 3 × s.d. Significance was assessed by Mann–Whitney U test on replicate samples.

### RESULTS

### *Reactivity against autologous cells*

Lymphocytes from 47 patients were tested for cytotoxicity against autologous

TABLE I.—*Cytotoxicity of lymphocytes from different sites against autologous tumour*

| Patient no. | Cytotoxicity in effectors from PBL | Near node | Far node | TIL |
|---|---|---|---|---|
| Duke's Stage B (12/24 show cytotoxicity against autologous tumour in at least one population) | | | | |
| 7 | 11·4* | 0 | — | — |
| 11 | 19·9* | 14·5* | 18·3* | — |
| 13 | 10·2* | 2·0 | 4·0 | — |
| 15 | 19·4* | 0 | 0 | — |
| 17 | 10·3* | 12·0* | — | — |
| 24 | 0 | 17·0* | 0 | 8·5 |
| 25 | 0 | 37·1* | — | — |
| 26 | 17·6* | 6·5 | 6·2 | — |
| 36 | 14·9* | 8·7 | 19·2* | — |
| 40 | 12·2* | 21·1* | 0 | 0 |
| 49 | 19·5* | 13·2* | 20·0* | — |
| 54 | 12·3* | — | — | — |
| Duke's Stage C (8/15 show cytotoxicity) | | | | |
| 1 | 0 | 20·0* | 0 | — |
| 9 | 13·7* | 8·0* | 16·7* | |
| 19 | 19·3* | 11·3* | 16·0* | — |
| 22 | 50·4* | 31·6* | 15·2* | 5·9 |
| 31 | 19·9* | 19·7* | 14·3* | 11·9* |
| 38 | 9·1* | — | 1·9 | 10·9* |
| 50 | 24·1* | — | 41·4* | 72·6* |
| 57 | 15·2* | 15·9* | 5·4 | 10·4* |
| Duke's Stage C+Metastases (3/7 show cytotoxicity) | | | | |
| 2 | 8·5* | 0 | 8·4* | — |
| 12 | 11·1* | 13·1* | | — |
| 60 | 21·0* | 14·8* | 14·4* | 17·5* |

* $P < 0{\cdot}05$.

TABLE II.—*Cytotoxicity of lymphocytes from different sites against autologous colon-tumour cells*

| Duke's stage | Frequency of reactive effectors from: (Percentages in parentheses) PBL | Near node | Far node | TIL |
|---|---|---|---|---|
| A | 0/1 | 0/1 | 0/1 | — |
| B | 10/22 (45) | 6/23 (26) | 3/17 (18) | 0/5 (0) |
| C | 7/14 (50) | 6/12 (50) | 5/13 (38) | 4/6 (67) |
| C+Mets | 3/7 (43) | 2/7 (29) | 2/5 (40) | 1/3 (33) |
| Total | 20/44 (45) | 14/43 (33) | 10/36 (28) | 5/14 (36) |

colon-tumour cells. Duke's staging of these patients showed one Stage A, 24 Stage B, 15 Stage C and 7 Stage C with distant metastases. Overall, 23/47 patients showed significant lytic activity in at least one lymphocyte preparation. The frequencies of positive reactions for PBL, near nodes, far nodes and TIL were 20/44, 14/43, 10/36 and 5/14 respectively. Data from these positive cases are presented in Table I and summarized in Table II. Positive cytotoxic reactions ranged from 8 to 72·6%. In most cases, several effector populations from individual patients showed reactivity against autologous cells. In 5 patients (7, 13, 15, 26, 54) lytic potential was limited to blood, and in 3 (24, 25, 1) to node. All others (15 cases) had cytotoxicity in 2–4 effector samples. Several cases showed widespread lytic activity through the different lymphocyte populations. This is particularly noticeable in Stage C. Cytotoxicity, when detectable, was dose dependent over the range tested (10:1–50:1). Only the higher ratio is presented in Table I.

In comparing the number of positive cases in different stages of disease, there was little evidence of association of reactivity with disease stage, though the low number of cases makes useful comparison difficult. Similarly, no significant difference in frequency of reactivity in different sites was apparent, except in TIL in Stage B, where no positives were recorded. Reactive TIL samples occurred in Stage C (4/6 positive) and C with distant metastases (1/3 positive).

Preliminary attempts to characterize the cytotoxic effectors were made by enrichment of T lymphocytes by passage through nylon columns (Julius *et al.*, 1973) or separation of cells forming rosettes with sheep red blood cells (SRBC) (Jondall *et al.*, 1972). Both methods enriched T cells from ~60% in unseparated samples to >85%. In almost all cases greater cytotoxicity for autologous colon-tumour cells was found in T-enriched populations (Fig. 1) and in 7 cases significant reactivity was induced in T-enriched preparations from previously unreactive samples. In view of these data, samples were separated by passage through nylon columns and SRBC rosetting whenever sufficient material was available, and it is the enhanced values for cytotoxicity in T-enriched populations that are shown in Table I. Enrichment of effector function

FIG. 1.—Enrichment of cytotoxic activity of PBL for autologous colon-tumour cells by (a) passage through nylon columns or (b) separation of SRBC rosette-forming cells. Effector:target ratio 40:1.

was apparent in both PBL (Fig. 1) and LNC (data not shown).

*Cytotoxicity against allogeneic tumour*

Specificity studies were performed by: (1) Testing the lymphocytes from colon-cancer patients against allogeneic colon-tumour cells; or (2) Testing lymphocytes from healthy donors against colon cancer (Table III). Blood lymphocytes from 12 healthy donors and 6 lung-tumour patients were tested, and 4 positive reactions against allogeneic colon cells recorded (3 with healthy donors). In all 4 of the cases effectors autologous with the target cells were also positive. The exact nature of the cytolytic interaction in these 4 cases has not been fully characterized. Lymphocytes from autologous LN were also cytotoxic, but did not kill K562. Levels of such "inappropriate" kill by the 4 reactive control donors (12·7–22·4%) approached those found in autologous combinations. Two of the 4 positive controls killed a range of targets including colon-carcinoma and lung-carcinoma cells, and cytotoxicity was again concentrated in nylon-column-

TABLE III.—*Specific and non-specific cytotoxicity in colon carcinoma*

| | Frequency of significant cytotoxicity against (Percentages in parentheses) | | |
|---|---|---|---|
| Effectors | Autologous colon-tumour cells | Allogeneic colon-tumour cells | K562 |
| PBL | 20/44 (45) | 2/18 (11) | 31/36 (86) |
| Near node | 14/43 (33) | 1/14 (7) | 6/37 (16) |
| Far node | 10/36 (28) | 0/11 (0) | 4/32 (13) |
| TIL | 5/14 (36) | 0/1 | 2/12 (17) |
| Control PBL | — | 4/18 (22) | 14/15 (93) |

FIG. 2.—Blocking of cytotoxicity of PBL for autologous tumour by admixture of unlabelled cells. Effectors and targets (E:T 20:1) were mixed in Luckham LP3 tubes and increasing numbers of unlabelled autologous (○) or allogeneic (●) colon-tumour cells added prior to centrifugation. * Significant reduction of cytotoxicity.

passed SRBC-rosetting cells. When colon-cancer patient's lymphocytes were tested against allogeneic targets, two positive reactions were found in blood and one reaction in nodes, in a total of 18 cross tests. The target cells in these cases were susceptible to autologous killing. The number of TIL was limited so that it was possible to test only one of these against control cells. As an additional specificity control, the capacity of unlabelled tumour cells to interfere with killing of autologous tumour or K562 was tested. Blocking of cytotoxicity against autologous tumour was found only with the autologous tumour (Fig. 2). Allogeneic cells were inactive in these cold inhibition assays. Similarly colon-carcinoma cells did not block lysis of K562 by lymphocytes from colon-cancer patients or healthy controls under conditions where unlabelled K562 gave significant dose-dependent inhibition of lysis.

FIG. 4.—Cytotoxicity of lymphocytes from different sites from patients with Duke's Stage B colon carcinoma against K562 cells. Mean reactivity ± s.d. is presented. Effector: target ratio 40:1.

*Cytotoxicity against K562*

All effector populations were tested for cytotoxicity against the K562 cell line, in order to determine the levels of NK potential. The lysis of K562 targets was apparent in most PBL samples from both healthy donors (14/15, 93%, reactive) and colon-cancer patients (Table III) (31/36, 86%, reactive) though the levels of killing were extremely variable (2–65% in controls and 0–64% in patients). Comparison of the cytotoxic potential of lymphocytes from patients with different stages of disease and controls revealed no deficit of activity in any group (Fig. 3). In healthy donor and disease groups the mean reactivity and range of results was closely similar, and no significant differences were found. By contrast, lymphocytes from draining nodes were only rarely cytotoxic for the cell line, as were those isolated from the tumour mass. All data for Stage B cases are presented in Fig. 4, where identical patterns of reactivity are seen in Stage C and C + metastases. In the total series of 69 nodes from 38 patients, 10 significantly reactive samples were recorded, but levels of cytotoxicity, even in positive nodes, were less than those achieved with PBL from the same individual. The reactivity of nodes was not related to disease stage (1A, 5B, 3C and 1C with metastases) distance from tumour (6 near, 4 far) or node histology (2 sinus histocytosis, 4 tumour, 4 normal). In all

FIG. 3.—Cytotoxicity of PBL from healthy donors and cancer patients of different Duke's staging for K562 cell line. Numbers beside columns show mean ± s.d. No significant difference between groups was found by *t* test Effector: target ratio 40:1

FIG. 5.—Correlation of cytotoxicity at an effector: target ratio of 40:1 against autologous tumour and the K562 cell line. $r = 0{\cdot}10$; $P > 0{\cdot}1$.

cases only one node was reactive; other nodes from the same patient failed to significantly damage K562. Nodes positive against K562 showed no greater reactivity against autologous tumour than negative groups. Subpopulation analysis was performed on 7 of these positive LNC. In all 5 tested, greatest cytotoxic potential resided in the SRBC-rosetting preparation. Two further samples separated on nylon columns showed enrichment for cytotoxicity in the adherent cells. TIL were cytotoxic in 2/12 cases (both Stage C), which were not related to levels of cytotoxicity in blood or lymph node.

Since effectors from blood were frequently reactive against both autologous tumour and K562, comparison was made to determine whether levels of killing were related (Fig. 5). Data were available for 36 PBL samples, but no significant correlation between killing of the two parameters was found (correlation coefficient = 0·10, $P > 0{\cdot}1$). PBL from the 4 healthy donors showing reactivity against colon-carcinoma cells did not have unusually high levels of NK activity as measured by lysis of K562. Additional evidence for the independence of the two cytotoxic phenomena was available from effector-cell separation studies similar to those described above. Passage of cells through nylon columns had little effect on cytotoxicity in patients showing significant augmentation of cytotoxicity against autologous cells. In a further series, highest killing of K562 was in populations from PBL which did not rosette with SRBC.

### DISCUSSION

In this paper we have delineated two major lymphocyte effector populations with activity against colonic-tumour cells: a relatively specific killer population, detectable in blood, lymph node and within the tumour itself, which is preferentially reactive with autologous cells; and a non-specific killer (NK) population, largely limited to blood, displaying high reactivity against the K562 cell line. The two effector types appear to be concentrated in lymphocyte populations with different cell-surface-marker characteristics, and to mediate mutually independent lytic interactions with appropriate sensitive targets.

The pursuit of specific disease-related cytotoxicity against colon carcinoma has received considerable attention, since first reports of selective killing of allogeneic colon-carcinoma cell lines by lymphocytes from colon-carcinoma patients (Baldwin *et al.*, 1973; Hellström *et al.*, 1970, 1971; Nairn *et al.*, 1971). With increasing awareness of the pervasive nature of spontaneously cytotoxic NK cells, particularly in long-term microcytotoxicity assays, the validity of early claims has been questioned (Takasugi *et al.*, 1973). Our own studies in this neoplasm are representative of those of many laboratories, where extensive testing against short-term cultures, using a variety of normal and pathological controls, failed to identify patterns of cytotoxicity which were unique to the colon-cancer group (Vose *et al.*, 1975). In comparing the present study with earlier investigations, several potentially important differences are apparent, not least of which is the nature of the target cells. Those used here were derived by the sequential application of separative techniques, exploiting differences in size, density and adherence properties of tumour components. By these means, cell selection, which is invariably associated with adaptation to tissue culture, and the concomitant induction of sensitivity to NK-mediated lysis (Becker *et al.*, 1978) were obviated. Extensive testing of similar freshly derived tumour-cell suspensions has shown that they are largely refractory to NK-mediated lysis and minimally express the relevant recognitive structure (Vose & Moore, 1980). As such they share with the short-term cultures of Nairn and associates (Nairn *et al.*, 1971; Pihl *et al.*, 1976) the property of permitting the measurement of specific effector function (Werkmeister *et al.*, 1979). In addition, killing was largely confined to autologous combinations in short-term assays. The preference shown for autologous cells under such conditions, together with the characteristics of the effector cells as well as the concentration of effectors (*i.e.* enrichment of cytotoxic efficacy by formation of SRBC rosettes and passage through nylon columns), support the interpretation that the primary mediators are T cells. This conclusion is strengthened by the frequency with which effectors were detectable in $NK^-$ LNC and TIL preparations, though the PBL compartment had the highest frequency of reactivity. There were no indications of accumulation of antigen-sensitized cells at the tumour site, in that only 3 individuals showed draining-node reactivity in the absence of cytotoxicity in the blood. TIL were almost invariably poorer effectors than those from other sites. Factors influencing TIL activity may be manifold, and include handling and the presence of substances inhibitory of lymphocyte function in the micro-environment of the neoplasm, a recent indication of which is the claim that repeated washing of TIL may restore their lytic potential (Hutchinson *et al.*, 1981). *In situ* there is evidence that these cells have suppressor activity, and are less responsive to mitogens than PBL (Vose & Moore, 1979). With regard to the lack of NK in LNC and TIL, unpublished observations have shown that these populations lack the large granular lymphocytes which mediate natural killing (Timonen *et al.*, 1981).

NK cells show spontaneous cytotoxicity against a broad range of target cells, including autologous lymphoblastoid cell lines (Herberman & Holden, 1978; Leibold & Peter, 1978). In the present study the activity of all populations against the K562 cell line was monitored as a measure of their NK effector capacity. No correlation was apparent between levels of NK and cytotoxic T cells reactive with autologous targets. This was not unexpected in view of the refractory nature of the freshly isolated targets to NK and the largely $NK^-$ status of LNC and TIL, observations which in our view question the relevance of this effector function in control of established neoplasia.

Most PBL showed variable but significant activity against the cell line. In contrast with the results of others (Pross & Baines, 1976) no decrease in lytic potential was disclosed with advancing disease, even in patients with widespread metastases. Cytotoxicity was recorded in only a minority of LNC. Whilst no relationship was found between the presence of lymph-nodal NK and clinico-pathological stage, it is of interest that nodal NK showed different isolation characteristics from those found in the blood, being SRBC rosetting and nylon-column adherent. These data are similar to those described for NK effectors in axillary nodes from breast cancer (Eremin *et al.*, 1978).

The detection of reactivity against autologous tumour in LNC which are $NK^-$ supports the independent nature of the specific killer population, and offers a means by which disease-related reactivities may be investigated, without recourse to the tedious tumour-cell separation used here or extensive depletion of NK effectors from PBL samples (Bakacs *et al.*, 1978).

Lymphocytes from 3 healthy donors and one lung-carcinoma patient showed cytotoxicity for colon-carcinoma cells. The nature of this allogeneic interaction is unclear, though again the finding of autologous reactivity in this patient in $NK^-$ nodes suggests a target-cell susceptibility to lysis unrelated to NK. The possibility that such unrestrained killer activity may result from polyclonal stimulation of cytotoxic effector function (Bonnard *et al.*, 1978) similar to that when lymphocytes are grown in conditioned media containing T-cell growth factor (Interleukin 2) is currently under investigation. Lymphocytes from blood, lymph node and tumour can be cultured in TCGF medium and most frequently show high cytotoxicity for autologous and allogeneic targets, but not K562 (Vose & Moore, 1981). In this extensive series we have failed to detect any correlation of specific and non-specific lysis of colonic tumour cells with the clinical course of the disease, in so far as anti-tumour reactivity was present in all clinico-pathological groups. The role of NK and cytotoxic T cells in control or progression of malignant disease therefore remains obscure. The data call for clonal expansion of the cytotoxic T-cell population expressing preferential reactivity with autologous tumour cells. The availability of such cellular reagents would permit a more exhaustive analysis of tumour specificity, and should help to determine their biological role.

This study was supported by grants from the Cancer Research Campaign and the Medical Research Council of Great Britain. We are indebted to Mr R. Ferguson for skilled technical assistance.

### REFERENCES

ANDERSSON, L. C., NILSSON, K. & GAHMBERG, C. G. (1979) K562 human erythroleukaemic cell line. *Int. J. Cancer*, **23**, 143.

BAKACS, T., KLEIN, E. & LJUNGSTROM, K. K. (1978) Search for disease-related cytotoxicity in mammary tumour patients. *Cancer Lett.*, **4**, 191.

BALDWIN, R. W., EMBLETON, M. J., JONES, J. S. P. & LANGMAN, M. J. S. (1973) Cell-mediated and humoral immune reactions to human tumours. *Int. J. Cancer*, **12**, 73.

BECKER, S., KEISSLING, R., LEE, N. & KLEIN, G. (1978) Modulation of sensitivity to natural killer cell lysis after *in vitro* explanation of a mouse lymphoma. *J. Natl Cancer Inst.*, **61**, 1495.

BONNARD, G. D., SCHENDEL, D. J., WEST, W. A. & 7 others (1978) Continued growth of normal human T lymphocytes in culture with retention of important functions. In *Human Lymphocute Differentiation. Its Application to Human Cancer.* (Eds. Serrou & Rosenfeld). Amsterdam: North Holland. p. 319.

EMESON, E. E. (1978) Migratory behaviour of lymphocytes with specific reactivity to alloantigens. *J. Exp. Med.*, **147**, 13.

EREMIN, O., COOMBS, R. R. A., PLUMB, D. & ASHBY, J. (1978) Characterisation of human natural killer (NK) cell in blood and lymphoid organs. *Int. J. Cancer*, **21**, 42.

HELLSTRÖM, T., HELLSTRÖM, K. E., PIERCE, G. E. & YOUNG, J. P. S. (1970) Cellular immunity to colonic carcinomas in man. In *Carcinoma of the Colon and Antecedent Epithelium.* (Ed. Burdette). Illinois: Thomas Springfield. p. 176.

HELLSTRÖM, T., HELLSTRÖM, K. E., SJÖGREN, H. O. & WARNER, G. E. (1971) Demonstration of cell-mediated immunity to human neoplasms of various histological types. *Int. J. Cancer*, **7**, 1.

HERBERMAN, R. B. & HOLDEN, H. T. (1978) Natural cell-mediated immunity. *Adv. Cancer Res.*, **27**, 305.

HUTCHINSON, G. H., HEINEMANN, D., SYMES, M. O. & WILLIAMSON, R. C. N. (1981) Differential immune reactivity of tumour-intrinsic and peripheral-blood lymphocytes against autoplastic colorectal carcinoma cells. *Br. J. Cancer*, **44**, 400.

JONDAL, M., HOLM, G. & WIGZELL, H. 1972. Surface markers on human T and B lymphocytes. I. A large population of lymphocytes forming non-immune rosettes with sheep red blood cells. *J. Exp. Med.*, **136**, 207.

JULIUS, M. H., SIMPSON, E. & HERZENBURG, L. A. (1973) A rapid method for the isolation of functional thymus derived lymphocytes. *Eur. J. Immunol.*, **3**, 645.

LOZZIO, C. B. & LOZZIO, B. B. (1973) Cytotoxicity of a factor isolated from human spleen. *J. Natl Cancer Inst.*, **50**, 535.

LEIBOLD, W. & PETER, H. H. (1978) Spontaneous cell mediated cytotoxicity (SCMC). Another immune defence system. *Behring Inst. Mitt.*, **62**, 144.

NAIRN, R. C., NIND, A. D. D., GULI, E. P. G. & 4 others (1971) Immunological reactivity in patients with carcinoma of colon. *Br. Med. J.*, iv, 706.

PERLMANN, P. & CEROTTINI, J-C. (1979) Cytotoxic lymphocytes. In *The Antigens*, Vol. 5 (Ed. Sela). New York: Academic Press. p. 173.

PIHL, E., NAIRN, R. C., NIND, A. P. & 4 others (1976) Correlation of regional lymph node *in vitro* anti-tumour immunoreactivity histology with colorectal carcinoma. *Cancer Res.*, **36**, 3665.

PROSS, H. F. & BAINES, M. G. (1976) Spontaneous human lymphocyte mediated cytotoxicity against tumour target cells. I. The effect of malignant disease. *Int. J. Cancer*, **18**, 593.

TAKASUGI, M., MICKEY, M. R. & TERASAKI, P. I. (1973) Reactivity of lymphocytes from normal persons on cultured tumour cells. *Cancer Res.*, **33**, 2898.

TIMONEN, T., ORTALDO, J. R. & HERBERMAN, R. B. (1981) Characteristics of human large granular lymphocytes and relationship to natural killer and K cells. *J. Exp. Med.*, **153,** 569.

VOSE, B. M. (1981) Separation of tumour and host cell populations from human neoplasms. In *Methodological Surveys,* Vol. 11 (Ed. Reid). Chichester: Ellis Harwood.

VOSE, B. M. & MOORE, M. (1979) Suppressor cell activity of lymphocytes infiltrating human lung and breast tumours. *Int. J. Cancer,* **24,** 579.

VOSE, B. M. & MOORE, M. (1980) Natural cytotoxicity in humans: Susceptibility of freshly isolated tumour cells to lysis. *J. Natl Cancer Inst.*, **65,** 257.

VOSE, B. M. & MOORE, M. (1981) Cultured human T cell lines kill autologous solid tumours. *Immunol. Letters* (In press).

VOSE, B. M., MOORE, M., SCHOFIELD, P. F. & DYMOCK, I. W. (1975) Leucocytotoxicity in malignant and non-malignant colonic diseases. *Clin. Exp. Immunol.*, **22,** 393.

VOSE, B. M., VANKY, F. T., ARGOV, S. & KLEIN, E. (1977) Natural cytotoxicity in man: Activity of lymph node and tumour infiltrating lymphocytes. *Eur. J. Immunol.*, **7,** 753.

WERKMEISTER, J. A., PIHL, E., NIND, A. A. P., FLANNERY, G. R. & NAIRN, R. C. (1979) Immunoreactivity by intrinsic lymphoid cells in colorectal carcinoma. *Br. J. Cancer,* **40,** 839.